# Hypogonadotropic Hypogonadism and Juvenile Idiopathic Arthritis in an African Boy: What is the Pathophysiological Link?

**DOI:** 10.7759/cureus.11337

**Published:** 2020-11-05

**Authors:** Ngoné Diaba Diack, Nafy Ndiaye, Aminata Mbaye, Baidy Sy Kane, Abdoulaye Leye

**Affiliations:** 1 Internal Medicine and Endocrinology, Pikine Teaching Hospital, Dakar, SEN; 2 Diagnostic Radiology, Aristide Le Dantec Teaching Hospital, Dakar, SEN; 3 Internal Medicine, Aristide Le Dantec Teaching Hospital/Cheikh Anta Diop University, Dakar, SEN

**Keywords:** male hypogonadotropic hypogonadism, systemic juvenile idiopathic arthritis, juvenile chronic arthritis, rheumatic diseases, africa

## Abstract

The association between hypogonadotropic hypogonadism and juvenile chronic arthritis has rarely been reported in the literature. We report an untreated case of systemic juvenile idiopathic arthritis in a young African male with co-presentation of hypogonadotropic hypogonadism. Possible pathophysiological and etiological links are discussed.

A 16-year-old boy was received in our outpatient department for chronic arthritis with temporomandibular involvement and fever. There was no family history of rheumatic diseases or psoriasis. Body temperature was 39.5°C at admission. The clinical examination found synovitis of wrists and knees and inflammatory lymphadenopathy. This polyarthritis occurred in a context of hypogonadism marked by impuberism of Tanner classification stage P2G2. Laboratory tests showed biological inflammatory syndrome and hyperferritinemia with collapsed glycosylated ferritin at 11%. Hormonal testing found low blood testosterone (0.08 mg/L) and pituitary hormone levels attesting to hypogonadotropic hypogonadism. Screening for infections was negative. The immunological assessment for antinuclear antibodies, rheumatoid factor, and anti-cyclic citrullinated peptide antibodies were negative. Standard radiography showed bilateral wrist carpitis. The olfactory bulb was present and normal by cerebral magnetic resonance imaging. The diagnosis of systemic juvenile idiopathic arthritis associated with hypogonadotropic hypogonadism, probably related to delayed puberty, was retained. A therapy combining corticosteroid, methotrexate for arthritis, and hormone replacement with testosterone led to regression of arthritis, biological inflammatory syndrome, and hypogonadism.

The presence of rheumatic disease in this context of hypogonadism, regardless of its cause, is mainly associated with very low testosterone levels and the presentation of arthritis in these patients tends to be more severe.

## Introduction

Hypogonadism refers to a clinical syndrome caused by a disruption at any level of the hypothalamic-pituitary-gonadal axis [1]. Male hypogonadism can result from a primary testicular disorder (primary hypogonadism) or can occur secondary to hypothalamic-pituitary dysfunction (hypogonadotropic hypogonadism). Although it is a common endocrine disorder, the exact prevalence of this disease is unknown [2].

Morbidity for men with hypogonadism includes decreased quality of life-related to sexual health, infertility, and an increased risk of developing acute and chronic illnesses [1,3]. Hypogonadism associated with rheumatic/autoimmune diseases is becoming more recognized and can occur as a result of the illness itself or its treatment. The presence of rheumatic/autoimmune disease in these patients is independent of the etiology of their hypogonadism and is associated with marked gonadal failure and consequent reduction of testosterone levels [4].

Hypogonadism is prevalent in patients with rheumatoid arthritis or lupus. A few studies have reported that low serum testosterone is predictive of seronegative arthritis [5]. However, the association between hypogonadotropic hypogonadism and juvenile chronic arthritis is rare [4,5]. Here we report an untreated case of systemic juvenile idiopathic arthritis (sJIA) in a young African male who also presents hypogonadotropic hypogonadism. The pathophysiological and etiological links between these two conditions are discussed.

## Case presentation

**Figure 1:**
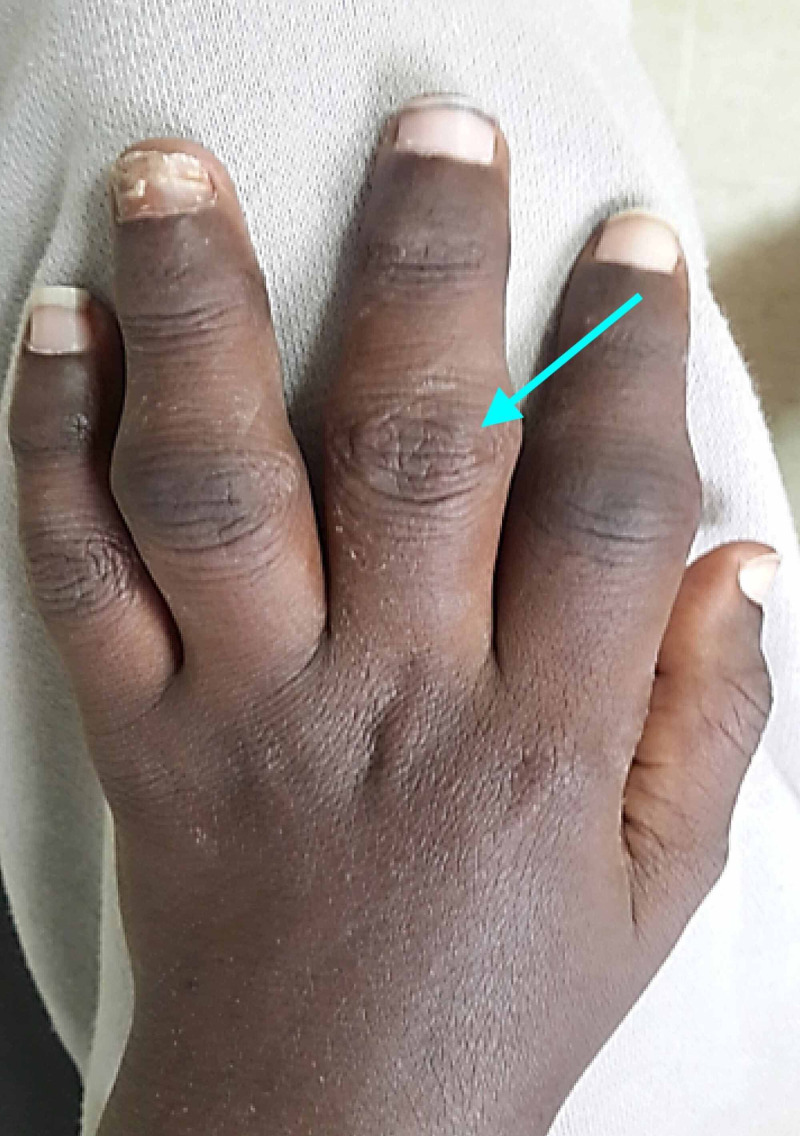
Synovitis in the patient's hand Inflammatory tumefaction of the metacarpophalangeal joints

A 16-year-old boy was received at the outpatient department with a history of intermittent fever and inflammatory arthralgia. The arthritis appeared over a period of three years and was evolving by thrust. Arthritis appeared symmetrically within the metacarpal joints of the upper extremities, the phalangeal joints of the wrist, and knees. This condition worsened gradually with occurrence of temporomandibular arthritis which induced permanent constriction of the jaws. This patient had no history of diarrhea. A family history of rheumatic diseases and psoriasis was not found. At admission, the patient had a temperature of 39.5°C, heart rate of 120 beats/minute, blood pressure of 120/90 mmHg, and breathing rate of 24 cycles/minute. A musculoskeletal examination showed synovitis of his wrists and knees (Figure 1). The clinical examination also found multiple enlarged lymph nodes (inflammatory lymphadenopathy). He had no sacroiliitis, enthesitis, or rash.

**Figure 2:**
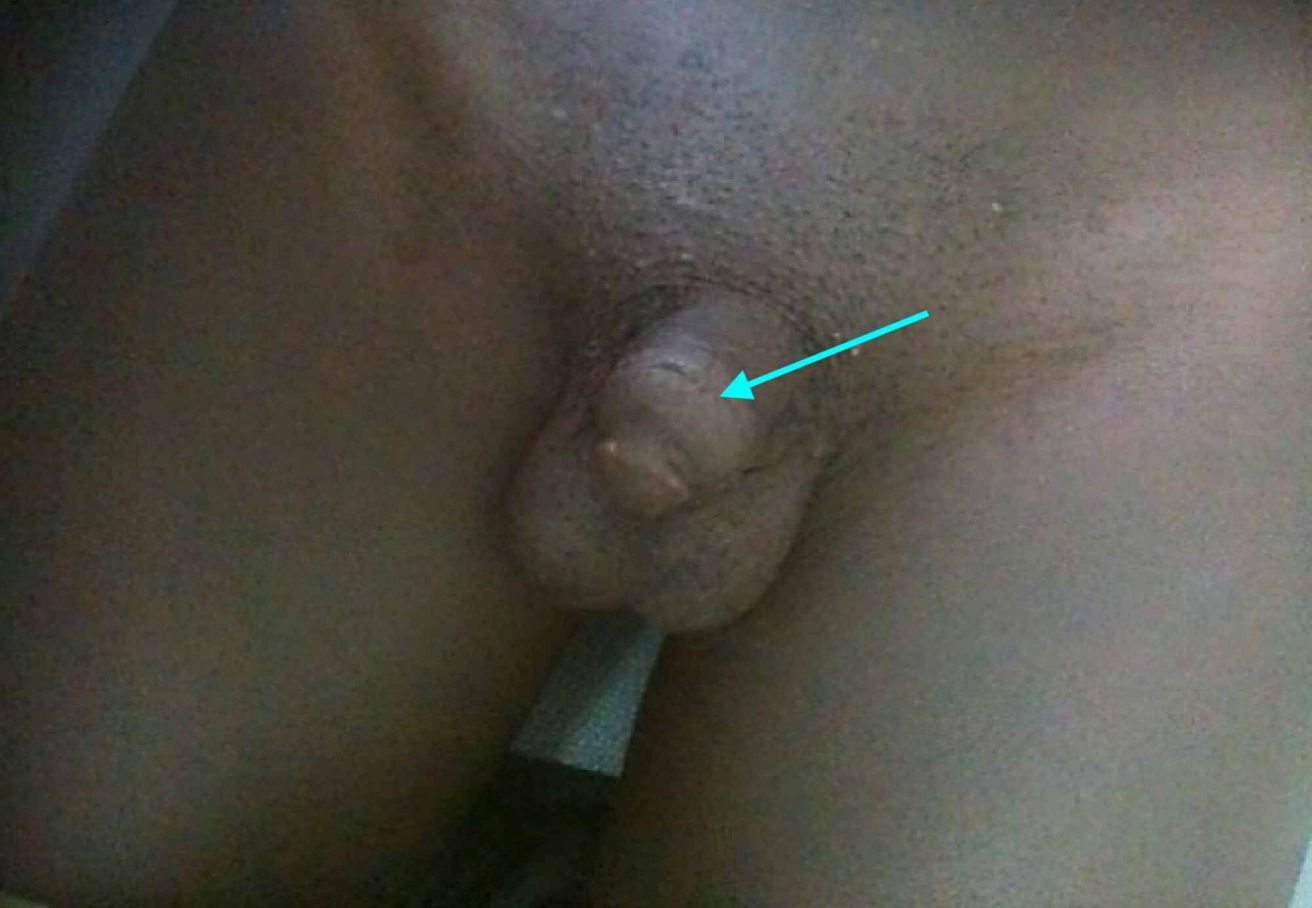
Impuberism with micropenia (Tanner stage P2G2)

This polyarthritis occurred in a context of hypogonadism marked by gynecomastia, a high-pitched voice, a delay in the development of secondary sexual characteristics, and pre-pubertal testicles Tanner classification stage P2G2 (Figure 2). He exhibited a failure to thrive characterized by a height of 145 cm, which corresponded with -2.5 standard deviation (SD) for his age, and a weight of 23 kg or -3 SD. The rest of the clinical examination was within normal limits; notably, there was no anosmia or hyposmia. 

Laboratory tests indicated a biological inflammatory syndrome and hyperferritinemia with collapsed glycosylated ferritin at 11%. The transferrin saturation was less than 20% (14.14%). Laboratory data is summarized in Table 1. Hormonal evaluation found low blood testosterone (0.08 mg/L) and pituitary hormone levels attesting to the hypogonadotropic hypogonadism diagnosis (Table 1). The other pituitary hormonal sectors were normal (Table 1). 

**Table 1:** Main Laboratory results recorded in our patient WBCs white blood cells, MCV mean corpuscular volume, MCHC mean corpuscular hemoglobin concentration, CRP C-Reactive Protein, N normal value, FSH Follicle Stimulating Hormone, LH Luteinizing Hormone, IGF1 insulin-like growth factor-1, TSH thyroid-stimulating hormone

	Laboratory results
Biological Inflammatory Syndrome	Neutrophilic leukocytosis : WBCs, 14.570/mm^3^ with neutrophils, 71,7%
	Anemia, hypochromic microcytic : hemoglobin, 8 g/dl ; MCH, 70.9 fl ; MCHC, 30.2g/dl
	Thrombocytosis : platelets, 619.000/mm^3^
	CRP = 107.5 mg/L (N< 5 mg/L)
	Hyperferritinemia 1286 ng/mL (5N)
Hormonal Exploration	Testosterone, 0.08 ug/l ê [N : 1,7-17,5]
	FSH, 0.2 UI/L ê [N : 2-11]
	LH, 0.5 UI/L ê [N : 1,7-8,6]
	IGF1, 85 ng/ml [N : 85,2-248,8]
	Prolactin, 13.04 ng/mL [N< 18]
	TSHus, 1.43 mUI/L [N : 0,5-4,7]
Immunological Assessment	Rheumatoid factor, negative
	Antinuclear antibodies, negative
	Anti- citrullinated peptide antibodies (ACPA), negative

Screening for infection included the *Plasmodium falciparum* thick blood test, blood cultures, cytobacteriological examination of urine, acid-fast bacillus test (for *Mycobacterium tuberculosis*), human immunodeficiency virus serology, and hepatitis B/C serology. The immunological assessment included profiling of antinuclear antibodies, rheumatoid factor, and anti-cyclic citrullinated peptide antibodies. All these tests were negative (Table 1).

**Figure 3:**
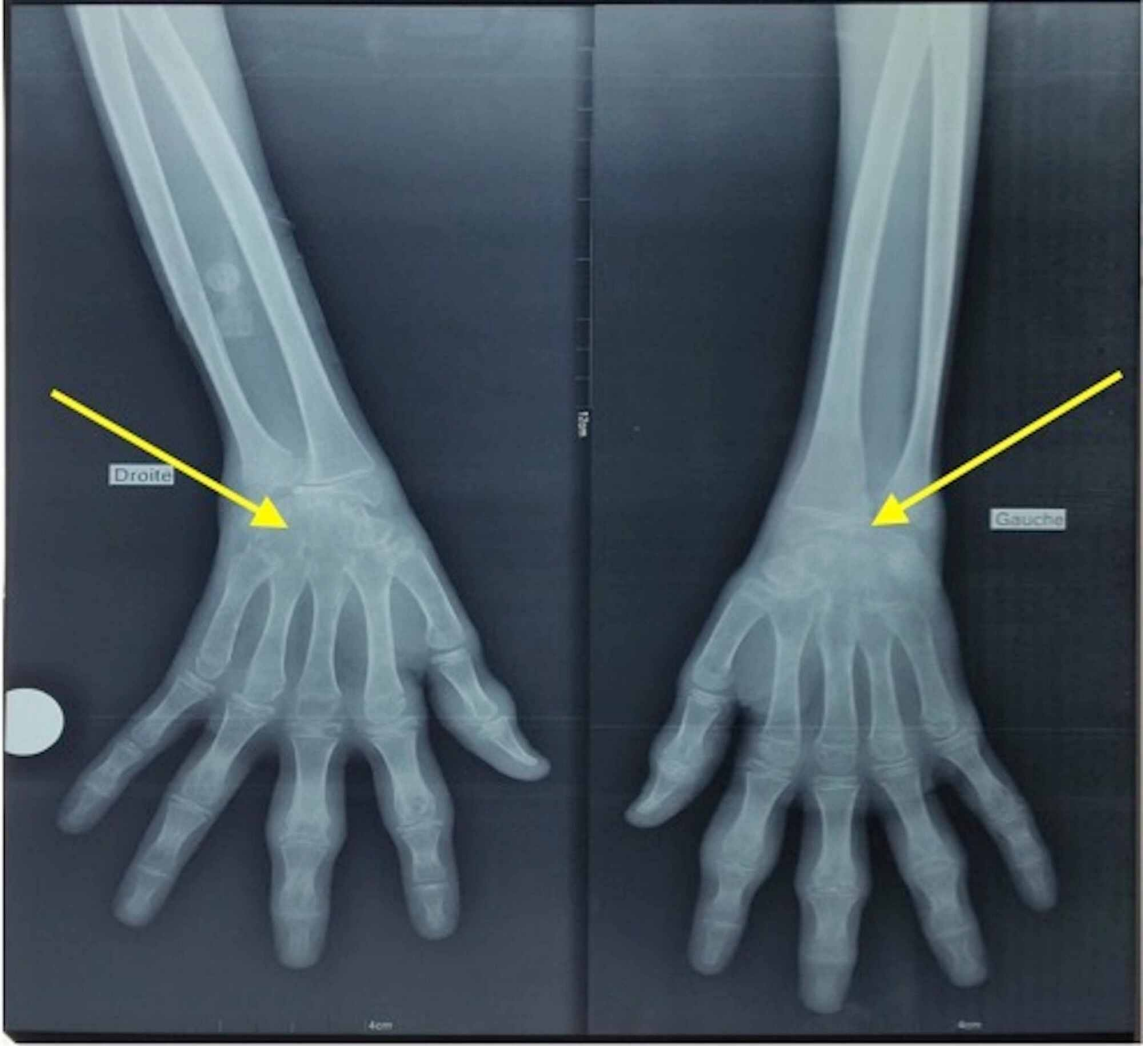
Bilateral wrist carpitis

Standard radiography showed bilateral wrist carpitis (Figure 3). 

**Figure 4:**
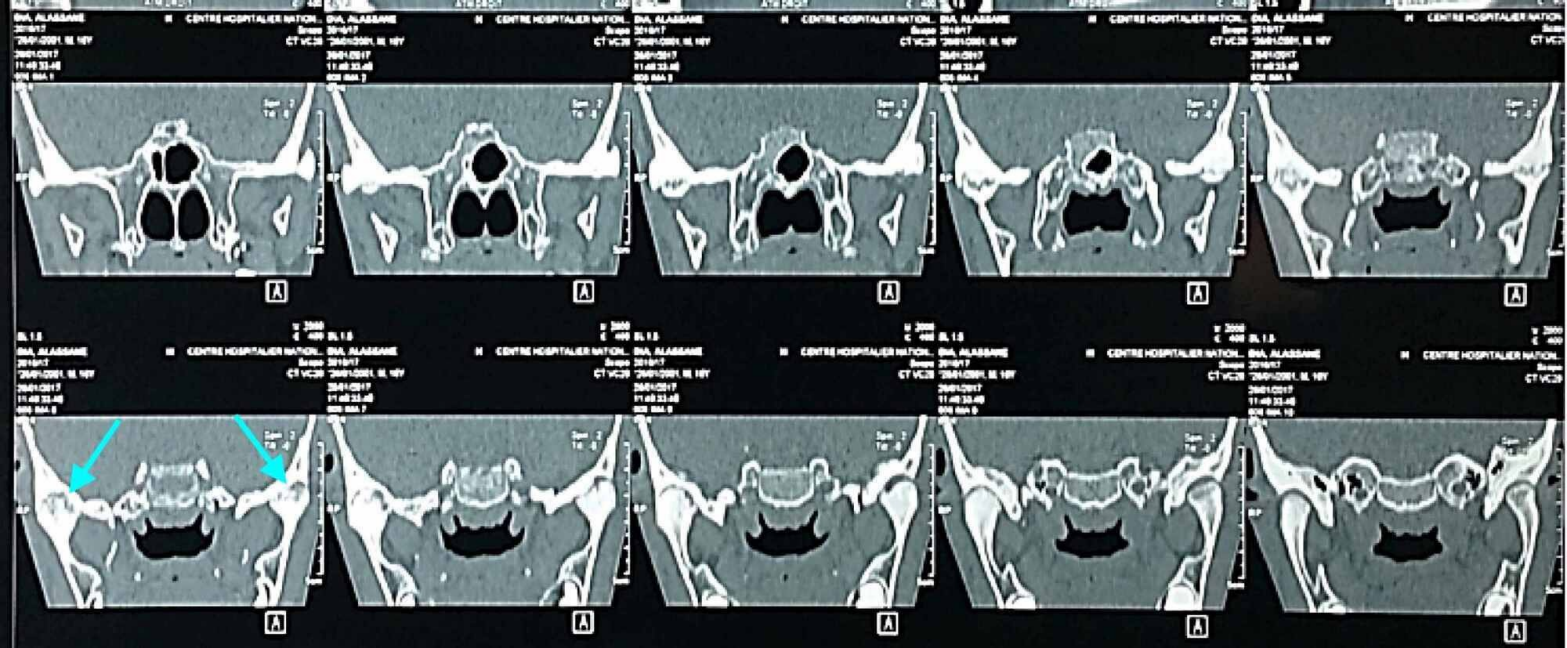
Temporomandibular arthritis Computed tomography showed destructive temporomandibular arthritis with bilateral ankylosis

Facial computed tomography showed destructive temporomandibular arthritis with bilateral ankylosis (Figure 4). 

**Figure 5:**
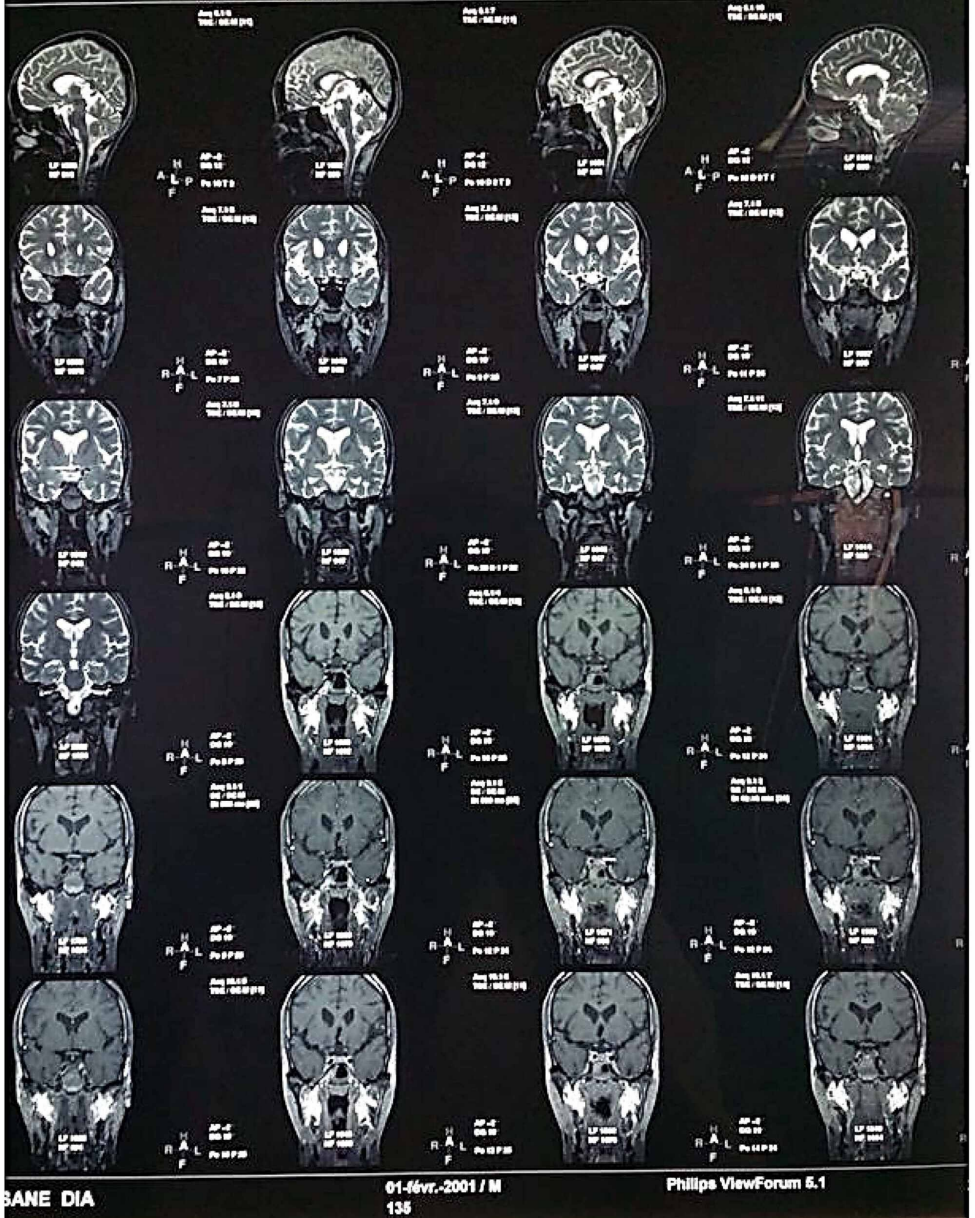
Cerebral magnetic resonance imaging of the patient

The olfactory bulb was present and normal by magnetic resonance imaging (Figure 5). There were no signs of hypophysitis. Furthermore, MRI found a pituitary microadenoma.

The diagnosis of sJIA associated of hypogonadotropic hypogonadism was retained. The activity of the disease was classified as high based upon the Juvenile Arthritis Disease Activity Score - 10 joints (cJADAS-10).

Initial treatment was based on corticosteroid therapy with prednisone at 1 mg/kg per day. The clinical course was marked by a clear improvement in the symptomatology, with stable apyrexia, arthritis regression (visual analog scale changed from 8/10 to 2/10), a C-reactive control protein level of 15 mg/L, and a hemoglobin level of 10.2 g/dl obtained after one week. For endocrine damage, hormone replacement with testosterone (injections of testosterone enanthate every three weeks) was used. On discharge, degression doses of corticosteroid was initiated after one month of treatment along with methotrexate at 25 mg/day. A follow-up three months later, with a good adherence and tolerance of the treatment (patient self-assessment), showed a complete regression of arthritis and biological inflammatory syndrome, and elevated residual testosteronemia. 

## Discussion

Hypogonadism associated with rheumatic/autoimmune diseases is becoming more recognized [3]. This study describes a case of sJIA associated with hypogonadotropic hypogonadism. This association has been the subject of limited research in the literature [4,5]. 

In terms of pathophysiology, this association could result from several mechanisms. Hypoandrogenism may play a role in rheumatic diseases and/or appear as a complication of a chronic inflammatory reaction [6]. Indeed, arthritis could be induced and/or promoted by low circulating testosterone levels. It has been shown that patients diagnosed with hypogonadism have an increased risk of developing any rheumatic/autoimmune disease more frequently than the general population [4,7]. The occurrence of arthritis in this context of hypogonadism appears regardless of its cause and is influenced mainly by marked gonadal failure with low testosterone levels. This was observed in our patient, who presented a very low testosterone level (0.08 mg/L) in accordance with a case reported in the literature [4,6]. 

The development of rheumatic/autoimmune diseases in patients with severe testicular dysfunction suggests that such abnormal function of the testes predisposes individuals to the development of rheumatic diseases [4]. Testosterone has anti-inflammatory effects via suppression of both the cellular and humoral immune systems. Testosterone deficiency has been shown to increase inflammatory markers, such as C-reactive protein, tumor necrosis factor, and interleukin-6 [7]. Thus, higher levels of testosterone may exert a protective role against rheumatic diseases [8]. However, more studies are needed to determine the exact mechanisms by which hypogonadism is interconnected with sJIA. 

Another possibility, which is not mutually exclusive, is that the association between hypogonadism and sJIA is related to the hypothalamic-pituitary localization of systemic diseases or the steroid therapies used in this context which are known to inhibit gonadotropin release from the pituitary [3,9]. 

The etiology of hypogonadism was not clearly identified in our patient. There was no previous steroid treatment and MRI found only a microadenoma. However, the latter alone is rarely responsible for hypogonadism [1,2]. Thus, even with the absence of signs of hypophysitis on MRI, cerebral localization of JIA in our patient could not be excluded in this context.

The clinical picture of rheumatic diseases in patients with hypogonadism is more severe, with major systemic and articular symptoms [4,10,11]. This was noted in our observation of destructive and ankylosis arthritis, an unusual presentation (temporomandibular involvement), and relatively high disease activity score [12]. Also, arthritis contributes to a decreased quality of life for patients with hypogonadism [1]. Therefore, it is important to start treatment quickly, especially when testosterone levels are very low, in order to avoid the induction of an underlying systemic condition that will negatively influence the prognosis and impair the lives of these patients. However, the treatment of hypogonadism in systemic disease is controversial and needs further study [3]. 

Although gonadotropin-releasing hormone or gonadotropin therapies are the best options for men wishing to have children, testosterone can be offered to patients who have systemic diseases for rapid symptomatic relief. Also, some studies have shown that testosterone replacement decreases inflammatory markers in these patients [7]. In our study, testosterone replacement combined with specific treatment for sJIA (corticosteroids + methotrexate) allowed symptoms to regress rapidly. The long-term effects of these treatments on fertility and growth remain to be clarified.

## Conclusions

Rheumatic/autoimmune diseases are a common problem in male patients with hypogonadism. However, the association between hypogonadotropic hypogonadism and juvenile chronic arthritis is rare. From our case study, it appears that the presence of rheumatic/autoimmune disease in this context of hypogonadism was mainly associated with very low testosterone levels and the presentation of arthritis was more severe.

Further studies are needed to determine the exact mechanisms by which hypogonadism is related to sJIA and the long-term effects of testosterone replacement on the prognosis of these patients. 

## References

[1] Richard-Eaglin A (2018). Male and female hypogonadism. Nurs Clin N Am.

[2] Fraietta R, Zylberstejn DS, Esteves SC (2013). Hypogonadotropic hypogonadism revisited. Clinics.

[3] Kalyani RR, Gavini S, Dobs AS (2007). Male hypogonadism in systemic disease. Endocrinol Metab Clin N Am.

[4] Jiménez-Balderas FJ, Tápia-Serrano R, Fonseca ME (2001). High frequency of association of rheumatic/autoimmune diseases and untreated male hypogonadism with severe testicular dysfunction. Arthritis Res.

[5] Perez-Garcia LF, Winkel B, Carrizales JP (2020). Sexual function and reproduction can be impaired in men with rheumatic diseases: a systematic review. Semin Arthritis Rheum.

[6] Lashkari M, Noori A, Oveisi S, Kheirkhah M (2018). Association of serum testosterone and dehydroepiandrosterone sulfate with rheumatoid arthritis: a case control study. Electronic Physician.

[7] Baillargeon J, AlSnih S, Raji MA (2016). Hypogonadism and the risk of rheumatic autoimmune disease. Clin Rheumatol.

[8] Tengstrand B, Carlström K, Hafström I (2009). Gonadal hormones in men with rheumatoid arthritis--from onset through 2 years. J Rheumatol.

[9] Salonia A, Rastrelli G, Hackett G (2020). Paediatric and adult-onset male hypogonadism. Nat Rev Dis Primers.

[10] Lee JY, Schneider R (2018). Systemic juvenile idiopathic arthritis. Pediatr Clin N Am.

[11] Consolaro A, Ruperto N, Bazso A (2009). Paediatric rheumatology international trials organisation. Development and validation of a composite disease activity score for juvenile idiopathic arthritis. Arthritis Rheum.

[12] Barut K, Adrovic A, Şahin S, Kasapçopur Ö (2017). Juvenile idiopathic arthritis. Balkan Med J.

